# Higher moment connectedness of cryptocurrencies: a time-frequency approach

**DOI:** 10.1007/s12197-023-09627-w

**Published:** 2023-05-22

**Authors:** Kingstone Nyakurukwa, Yudhvir Seetharam

**Affiliations:** School of Economics and Finance, University of the Witwatersrand, Johannesburg, South Africa

**Keywords:** Realised skewness, Realised kurtosis, High-frequency data, Risk spillovers, C58, G10, G15

## Abstract

The purpose of the study is to examine higher moment connectedness among 12 cryptocurrencies using data sampled at the 1-minute high-frequency interval. We use methods that demonstrate the heterogeneity of agents from their distinct investing horizons. This includes wavelet multiple cross-correlations, CEEMDAN-based Diebold-Yilmaz (DY) connectedness index and the Barunik-Krehlik (BK) frequency connectedness index. First, our results show that higher moment multiple correlations among the sampled cryptocurrencies are higher at all time scales and the relationship strengthens at lower frequencies. Second, the wavelet cross-correlations show different cryptocurrencies with the potential to lead and lag in the transmission of higher moment shocks to the whole system at different frequencies. Again, the multiple wavelet cross-correlations increase with increasing time scales. The results from the CEEMDAN-based DY connectedness index as well as the BK framework also reveal cyclical connectedness and differences in connectedness across different frequencies. The results show more connectedness of higher moments than the connectedness empirically reported for returns and volatility. Cryptocurrency connectedness has mostly been examined using the first two moments. We extend this line of literature by examining the third and fourth moments, which might be more useful for risk management purposes.

## Introduction

Traditional asset pricing models like the Capital Asset Pricing Model of Sharpe (1964) and Lintner (1965) are premised on the assumption of a Gaussian distribution of returns. The assumption that asset returns are normally distributed means that the risk-return properties of an asset or a portfolio of assets can be adequately described by the first two moments, i.e., mean and variance. In reality, however, financial assets often have stylised facts like leptokurticity and fatter tails. This departure from a Gaussian distribution means that the first two moments of the return distributions of financial assets may not be adequate for portfolio risk management and asset pricing (Ahmed and Al Mafrachi 2021) as there are increased chances of extreme negative returns. Cryptocurrency as an asset class has increased in popularity in recent times and extant literature has been devoted to its styled facts. According to Hasan et al. (2021), in the cryptocurrency market, higher moments like skewness and kurtosis are prominent because of the speculative trading that is driven by younger investors. However, existing literature so far has mainly concentrated on the first and second moments. In this study, we seek to fill this gap by examing the connectedness of the third and fourth moments among 12 selected cryptocurrencies.

The majority of the studies that have investigated the interconnectedness of cryptocurrencies have mainly focussed on the first two moments, namely, returns (e.g. Panagiotidis et al. 2018) and volatility (e.g. Stengos et al. 2022) and have mostly used wavelet analysis to untangle the connectedness of the assets (e.g. Bouri et al. 2020a, b). Kumar and Ajaz (2019) utilised wavelet-based methods to determine the extent of integration among Bitcoin, Ethereum, Lite and Dashcoin. The authors identified Bitcoin as a potential market leader using the first moment and that the wavelet multiple correlations among the cryptos follow an aperiodic cyclical nature. Qiao et al. (2020) investigated the time-frequency connectedness of cryptocurrency returns and volatility using wavelet analysis and reported that the volatility of returns is heterogeneous in a time-frequency space. Bitcoin was also reported as leading other cryptocurrencies in return and volatility spillovers and closely correlated with other cryptocurrencies.

Agyei et al. (2022) use multi-scale and time-frequency analysis to determine the degree of integration among six cryptocurrencies and the cryptocurrency-implied volatility index (VCRIX). The study reports high integration among the sampled cryptocurrencies. The VCRIX was also found neither leading nor lagging the interdependencies among the cryptocurrencies using the Wavelet Multiple Cross-Correlation. Qureshi et al. (2020) report that Ripple and Ethereum appear to have a significant influence on other cryptocurrencies (Ripple, Litecoin, and Bitcoin Cash), as indicated by the cross-wavelet transform. The results suggest that these two cryptocurrencies may be playing a contagion role in the market, spreading their effects to other cryptocurrencies. Dowling (2022) examines the potential linkages between the pricing of non-fungible tokens (NFTs) and cryptocurrencies, given that NFTs have emerged from the cryptocurrency market. The authors utilise a spillover index to investigate the extent of volatility transmission effects between the two markets. The results show limited evidence of such effects. However, through wavelet coherence analysis, the authors identify co-movements between the two markets, indicating a potential relationship between NFT and cryptocurrency prices. Related to this, Vidal-Tomás (2023) also report that the NFT prices are affected by cryptocurrency prices.

Other studies have concentrated on the evolution of cryptocurrency connectedness during the COVID-19 black swan. Kumar et al. (2022) employ the Baruník and Křehlík (2018) methodology (henceforth called the BK framework) to uncover the evolution of the connectedness among the 10 most capitalised cryptocurrencies from the pre-COVID-19 period to the COVID-19 period. The highlights of the study show that short-term spillovers dominate for considerable periods and that Ethereum is more influential than Bitcoin. The authors concentrated on the connectedness of volatility, the second moment and reported increased spillover effects during the COVID-19 period. Polat and Kabakçı Günay (2021) use a sample period that encompasses the period after the declaration of COVID-19 as a global pandemic to compute the short-term, medium-term and overall spillover indexes among cryptocurrencies using different frequency bands. The study reports that the spillover indexes computed captured the 2018 crypto market crash and the COVID-19 pandemic as linkages surged around these events. Shahzad et al. (2021) examine the daily return spillover among 18 cryptocurrencies under high and low volatility regimes. The study reports that the spillover among the cryptocurrencies is time-variant and that it is amplified around the first news of the COVID-19 pandemic, confirming the existence of high contagion in stress periods.

Li et al. (2020) use the Diebold and Yilmaz (2012) methodology (henceforth called the DY framework) to untangle the risk connectedness among 7 cryptocurrencies and find that the directions of risk spillovers are highly associated with the market capitalisations of the cryptos. The study reported that small-cap cryptocurrencies tend to transmit risks to large-cap cryptocurrencies. In a systematic review of related literature on return and volatility spillovers among cryptocurrencies, Kyriazis (2019) reports that Bitcoin is the most influential cryptocurrency and is a net transmitter and receiver of spillovers to and from other cryptocurrencies. The survey of literature also shows that Ethereum, Litecoin and Ripple are the most integrated cryptocurrencies with Bitcoin. Also, most of the studies surveyed showed that among cryptocurrencies, return spillovers were more pronounced than volatility spillovers.

While the focus of most of the studies outlined above has been on the intra-asset connectedness of cryptocurrencies, several other studies have attempted to establish the extent to which cryptocurrencies are connected with other asset classes. In a cross-asset analysis that included cryptocurrencies and commodity markets, Mo et al. (2022) report that during the COVID-19 period, the overall spillovers from cryptocurrencies to commodity markets varied over time. According to the spillover results in the time-frequency domain, cryptocurrencies played a significant role in transmitting risk to the system both in the short term and long term. Panagiotidis et al. (2019) reported that Bitcoin exhibits a strong connection with traditional stock markets, while its connection with foreign exchange markets and the broader macroeconomic environment is relatively weaker.

The majority of the studies reviewed above mainly used the first two moments, i.e. returns and volatility. Several studies reveal the inadequacy of the first and second moments in examining cryptocurrencies because of the departure from the Gaussian distribution. Studies have confirmed some stylised facts peculiar to cryptocurrencies, which Hasan et al. (2021) termed higher-moment-like features. The higher moment-like features postulate that the connectedness through the first and second moments may not be sufficient to fully understand the connectedness of cryptocurrency markets. Studies have documented a departure from Gaussian distribution in cryptocurrencies (e.g. Baek and Elbeck 2015), the lottery effect (e.g. Grobys and Junttila 2021) as well as the presence of asymmetry and tail risk (Cheikh et al. 2020). It is therefore essential to explore the connectedness of cryptocurrencies *via* higher moments.

Some studies have examined higher moment connectedness of financial assets, mostly across different asset classes. Bouri et al. (2022) examine the link between S&P500 and Bitcoin in higher-order moments, up to the fourth conditional moment, using the time-scale perspective of wavelet coherence analysis. The study uses data from 2011 to 2022, and the results indicate that the comovement between Bitcoin and S&P500 is moment-dependent and varies across time and frequency. The authors find that the comovement is more significant at mid and long-term scales for skewness and kurtosis. Overall, the study sheds light on the varying strength and nature of the relationship between Bitcoin and S&P500 in different moments and time scales. Gkillas et al. (2022) analyse spillovers in jumps and realised moments up to the fourth order among crude oil, gold, and Bitcoin markets using high-frequency data from 2014 to 2018. The authors employ Granger causality and generalised impulse response analyses to investigate the interdependence between the three markets. The results highlight the existence of predictability and underscore the importance of jointly modelling linkages across the three markets, especially with higher-order moments. Failure to do so may result in inaccurate risk assessment and investment inferences.

Bouri et al. (2021) focus on the spillover effects on realised estimators of return distributions among US stock, crude oil, and gold markets. Using 5-minute data from 2006 to 2019, the study computes daily realised volatility, realised skewness, realised kurtosis, and jumps. The authors employ a time-varying parameter vector autoregression (TVP-VAR) model to examine the dynamics of spillovers, taking into account various crisis periods. The findings reveal that spillovers intensify during crisis periods across all three markets. The study identifies the main net transmitter of spillovers for realised volatility and kurtosis to be the stock index, while for realized skewness and jumps, oil emerges as the primary transmitter.

Bouri et al. (2023) explore the connectedness in option implied moments, including volatility, skewness, and kurtosis, across precious metals (gold, silver) and energy (crude oil and natural gas) markets in both time and frequency domains. Daily option data from 2010 to 2020, is used to construct implied moments, and their static and dynamic connectedness is examined through time-frequency spillover methods and network analysis. The findings suggest that system-wide connectedness weakens as the moment order increases, and spillovers in all implied moments are more pronounced at lower frequencies. The intensity of spillovers varies over time and is particularly prominent during turbulent periods.

Nekhili and Bouri (2023) present evidence of the importance and usefulness of considering spillovers in volatility and higher-order moments (such as skewness and kurtosis) and co-moments (including covariance, co-skewness, and co-kurtosis), and their impact on hedging. The study employs a time-varying spillover approach and portfolio analysis using high-frequency data from the US stock, crude oil, and gold markets. The authors report that, in addition to volatility and covariance, co-skewness and co-kurtosis are significant spillover transmitters across these markets. This suggests that the skewness and kurtosis of one market can affect the others, emphasising the importance of understanding these spillovers for effective risk management and hedging strategies. The findings have practical implications for diversifying portfolios and employing dynamic hedging approaches that consider the changing spillover effects over time. The literature on higher moment connectedness of financial assets invariably uses cross-asset analyses. Some investors could be interested in diversification within a specific asset class, for example, cryptocurrency, hence the need to understand intra-class connectedness.

In this study, we examine the connectedness of cryptocurrencies using the third and fourth moments. In the domain of statistics, moments are scalar quantities that provide summative information concerning the univariate properties of the underlying data distribution. Moments give the average of various powers of deviations from the mean of unimodal distributions. The first (mean) and second (variance) moments quantify the location and dispersion of a distribution respectively. The third (skewness) and fourth (kurtosis) moments are used to identify the shape of a particular distribution. The skewness verifies the symmetry of distribution around the mean where a zero value implies symmetry while positive (negative) values imply asymmetry. A tendency toward positive skewness is a reflection of investors’ penchant for lottery-like assets (Bordalo et al. 2012) while negative skewness points to a source of crash risk (Jondeau et al. 2019) or tail risk (Bollerslev et al. 2015). On the other hand, the fourth moment shows the degree of flatness or peakedness of a distribution around its mean. A distribution with a kurtosis greater (lower) than three is described as being leptokurtic (platykurtic), with a thinner (flatter) peak, shorter (taller) shoulders and heavier (lighter) tails than the Gaussian distribution (Ahmed and Al Mafrachi 2021). A leptokurtic (platykurtic) distribution shows that extreme outcomes are more (less) likely to occur. The kurtosis is also known as the volatility of volatility (Ahmed and Al Mafrachi 2021) and reflects the presence of extreme values and outliers in the data.

According to recent research, the study of higher moment connectedness of cryptocurrency returns is important for understanding the behaviour of cryptocurrency markets and making informed investment decisions (Kristoufek 2018). Gourieroux et al. (1984) emphasise the importance of higher moment measures in capturing nonlinear dependencies in financial data. Bouri et al. (2020b) suggest that higher moment connectedness measures can help investors construct better-diversified portfolios that are less exposed to systemic risk in the cryptocurrency market. Additionally, higher moment connectedness measures can have regulatory implications, as they may suggest that certain cryptocurrencies are systemically important and in need of additional regulatory scrutiny.

Agents’ heterogeneity in financial markets as demonstrated by differences in beliefs, information, preferences, and behaviour among market participants can lead to diverse trading strategies, which can result in different levels of risk and return. The heterogeneity of agents in financial markets can contribute to the occurrence of higher moments in the distribution of returns. For example, if a group of investors has different beliefs about the prospects of a stock, their trading decisions can result in skewness in the distribution of returns. The presence of higher moments in financial markets can have significant implications for risk management, asset pricing and investment strategies. Understanding the impact of agents’ heterogeneity on the occurrence of higher moments is crucial in developing effective risk management and investment strategies. Additionally, considering the impact of higher moments and agents’ heterogeneity can help investors better understand the complexity and dynamics of financial markets.

Our study is close to Hasan et al. (2021) who investigated the high moment connectedness of cryptocurrencies using 5-minute high-frequency data. Using Diebold and Yilmaz’s (2012) spillover index, the authors report a robust skewness connectedness between Bitcoin and Ethereum. The study also reports a strong kurtosis connectedness between Bitcoin and Ethereum which peaks during the extreme phases of the COVID-19 pandemic. We expand this line of research by investigating the higher moment connectedness among selected cryptocurrencies in a time-frequency domain. Motivated by the with Heterogeneous Market Hypothesis (Müller et al. 1995) and the Fractal Markets Hypothesis (Peters 1994), we assume that contrary to the Efficient Markets Hypothesis (Fama 1965; Malkiel and Fama 1970), investors are not homogeneous but rather heterogeneous depending on their investment horizon. The heterogeneity of investors in the cryptocurrency market has been confirmed in some studies (e.g. Li et al. 2020). It is therefore of paramount importance to disaggregate the higher moment connectedness into different frequencies and across time to reflect the heterogeneity of investors.

Using Wavelet Multiple Correlation, we report high connectedness among the cryptocurrencies in both kurtosis and skewness in all frequencies and across time. Higher moment connectedness established from wavelet multiple correlations (WMC) increases with scale and peaks at close to 1 at the lowest frequency. The findings from the local CEEMDAN-based DY framework reveal that the higher moment connectedness among the cryptocurrencies is cyclical, albeit aperiodical at higher frequencies and smoothens at lower frequencies. Using the CEEMDAN-based DY framework and BK framework, we also establish that higher moment connectedness is especially high at lower frequencies.

The study proceeds as follows: in the next section, we look at the methodology used to examine the connectedness of cryptocurrencies, followed by a presentation of the results and the discussion thereof. The study ends with a conclusion that wraps up the study and provides potential areas for future studies.

## Data and methodology

### Data

We include data gathered at 1-minute granularity for twelve cryptocurrencies namely Bitcoin (BTC), Binance (BNB), Cardano (ADA), Ethereum Classic (ETC), Ethereum (ETH), Chainlink (LINK), Litecoin (LTC), Neo (NEO), Tron (TRX), Stellar (XLM), XRP (XRP), Zcash (ZEC). The 1-minute data are collected from the Binance exchange and downloaded from https://www.cryptodatadownload.com/data/. According to Alexander and Dakos (2020), using reliable data is crucial when assessing crypto market efficiency, portfolio optimisation, hedging, and trading opportunities. The authors argued that traded data from crypto exchanges should be used instead of data from coin-ranking sites to avoid the use of unreliable data. They recommended using traded prices from exchange platforms because coin-ranking sites compute a unique non-traded price using a methodology that may not accurately reflect market conditions. Our data source is also in line with comparable studies (e.g. Hasan et al. 2021). The sample data starts on 12 March 2020 at 0515 h and ends on 14 March 2022 at 0336 h together making 1 048 574 1-minute observations for each cryptocurrency. The choice for the start date and time as well as the end date and time was made solely based on the availability of minute-interval high-frequency data. The choice of cryptocurrencies was also chosen based on the availability of high-frequency data and the liquidity of the cryptocurrencies.

### Realised high moment

The ${i}^{th}$ intraday log return for each cryptocurrency on day $t$ is defined as:

1 $${r}_{t,i}=log\left(\frac{{P}_{t,i/N}}{{P}_{t,(i-1)/N}}\right)$$

Where P is the price of the cryptocurrency and $N$ is the number of return observations in a trading day. We use 1-minute returns so that in 24 trading hours in a day, we have  *N* = 1440. The opening log price on day *t* is P_t,0_ and the closing log price on day *t* is P_t,1._

In line with Amaya et al. (2015), we define ex-post realised daily skewness based on intraday returns standardised by realised variance as follows:

2 $${Rskew}_{t}=\frac{\sqrt{N}\sum _{i=1}^{N}{r}_{t,i}^{3}}{{Rvar}_{t}^{3/2}}$$

Realised daily kurtosis is defined following Amaya et al. (2015) again as follows:

3 $${Rkurt}_{t}=\frac{N\sqrt{N}\sum _{i=1}^{N}{r}_{t,i}^{4}}{{Rvar}_{t}^{2}}$$

The scaling of ${Rkurt}_{t}$ and ${Rskew}_{t}$ by $\sqrt{N}$ and $N$ ensures that their magnitudes correspond to daily skewness and kurtosis.

### Econometric model

#### Wavelet analysis

Though our ultimate objective is to demonstrate the spillover of higher moment return shocks using the seminal spillover indexes, we first establish the multiple correlations among the cryptocurrencies using wavelets. Simultaneous inclusion of methods based on wavelets and spillover indexes provides robustness in trying to understand the connectedness among variables and this approach has been used in recent studies (e.g. Al-Yahyaee et al. 2020). Wavelet analysis is increasingly being adopted in the domains of economics and finance to untangle the comovements and coherence of variables mostly in a bivariate framework. However, the bivariate wavelet correlation has been criticised because of several limitations and the Wavelet Multiple Correlation suggested by Fernández-Macho (2012) has been proffered as more succinct. First, the traditional pairwise wavelet approaches that utilise $n$ variables to estimate correlation and cross-relation lead to excessively high pairwise correlation coefficients ($n(n-1)/2)$ which may be difficult to accurately interpret. Also, Kumar Tiwari et al. (2013) emphasise the weakness of pairwise wavelet coefficients in a multivariate framework as possible relationships among the variables may lead to spurious coefficients. These and other weaknesses make Wavelet Multiple Correlation superior and clearer to interpret than the pairwise coefficients.

To examine the higher moment connectedness of the 12 cryptocurrencies sampled for this study, we depend on the Wavelet Multiple Correlation (WMC) of Fernández-Macho (2012). In computing the WMC, we first utilised the Maximal overlap Discrete Transform (MODWT) for each of the cryptocurrency skewness and kurtosis. In line with Fernández-Macho (2012), we preferred the MODWT over the Discrete Wavelet Transform (DWT) since the former has some documented advantages over the classical DWT. These advantages include the MODWT having a variance estimator that is asymptotically efficient compared to that of the DWT which makes the former more appropriate for computing wavelet correlations (Fernández-Macho 2012). Assuming that ${X}_{t}={x}_{1t}, {x}_{2t},\dots ,{x}_{nt}$ is a multivariate stochastic process and that ${W}_{t}={w}_{1jt}, {w}_{2jt},\dots ,{w}_{njt}$ is the corresponding scale ${\lambda }_{j}$ wavelet coefficients attained from the application of MODWT to each ${x}_{it}$ process, the WMC (defined as a single set of multiscale correlations) is computed as follows:

4 $${\varphi }_{X}\left({\lambda }_{j}\right)= \sqrt{1-\frac{1}{\text{max} \ diag \ {P}_{j}^{-1}}}$$

Where:

${P}_{j}$ refers to the $n\times n$ correlation matrix of ${W}_{jt}$ and the max diag (.) operator provides a selection for the largest element in the diagonal of the argument.

To identify a potential leader that can influence other cryptocurrencies within a system, we utilise the Wavelet Multiple Cross-Correlation (WMCC). WMCC can be used to identify potential leaders and followers in a set of time series data. This can be particularly useful in financial and economic applications where identifying the leader-follower relationships between different assets or indicators can provide insights into portfolio management and risk assessments. By applying WMCC to a set of time series data, we can analyse the strength and direction of the cross-correlations between different time series at different frequencies or scales. By allowing lag $\tau$ between the observed and the fitted values of the criterion variable at each scale ${\lambda }_{j}$, the WMCC is defined as:

5 $${\varphi }_{X,t}\left({\lambda }_{j}\right)=Corr\left({w}_{ijt}{\widehat{w}}_{ijt+\tau }\right)= \frac{Cov({w}_{ijt}, {\widehat{w}}_{ijt+\tau })}{Var\left({w}_{ijt}\right)Var\left({\widehat{w}}_{ijt+\tau }\right)}$$

Where:

${w}_{ijt}$ on the set of regressors $\left\{{w}_{kj}, k\ne j\right\}$ leads to the maximisation of the coefficient of determination ${\varphi }_{X}\left({\lambda }_{j}\right)$ and ${\widehat{w}}_{ij}$ is the fitted value of the regression.

#### CEEDMAN-based Diebold and Yilmaz framework

To examine the higher-order connectedness of the cryptocurrencies, we also depend on the seminal Diebold and Yilmaz (2012) spillover index. However, because we would like to examine how the higher moment connectedness of the cryptocurrencies evolves across different investing horizons to demonstrate the heterogeneity of investors, we decompose our variables into intrinsic time using the Complete Empirical Ensemble Mode Decomposition with Adaptive Noise (CEEMDAN) which is part of Huang (1998)’s Empirical Mode Decomposition (EMD) family. The decomposition of variables into intrinsic time using CEEMDAN helps in demonstrating the heterogeneity of investors. Specifically, we use five Inherent Mode Functions (IMFs) to represent intrinsic time with the lowest IMF (IMF 1) representing the highest frequency and the highest IMF (IMF 5) representing the lowest frequency. According to Liu et al. (2018), the CEEMDAN is an improvement on the EMD as it can seamlessly accommodate the processing of non-linear and non-stationary signals.

The computation procedure of the CEEMDAN is guided by Torres et al. (2011) and is implemented in the R package ‘Rlibeemd’ (Luukko et al. 2016). The initial step starts with a signal ${x}_{t}=s\left(t\right)+n\left(t\right)$ where $s\left(t\right)$ is the actual signal and $n\left(t\right)$ is the noise. Adding a Gaussian white noise ${\omega }_{k}\left(t\right)$ of distinct amplitudes to $x\left(t\right)$ results is various new signals:

6 $${x}_{k}\left(t\right)=x\left(t\right)+{\omega }_{k}\left(t\right)$$

The second step involves the deployment of the Empirical Mode Decomposition (EMD) to decompose Eq. (6) to obtain the first IMF. This is followed by estimating the average of this first IMF as follows:

7 $$\overline{{IMF}_{1}\left(n\right)} =\frac{1}{K}\sum _{k=1}^{K}{IMF}_{1k}\left(t\right)$$

This is followed by the computation of the remainder of the signal components ${C}_{n}$ of the original signal as

8 $${C}_{n}=\left\{\begin{array}{c}x\left(t\right)-{IMF}_{1}\left(n\right), n=1,\\ {C}_{n-1}-{IMF}_{1}\left(n\right), n>1.\end{array}\right.$$

The $\rho -th$ component post-EMD of the signal is denoted by ${E}_{\rho }$. Subsequent components (L + 1IMFs) are computed as follows:

9 $${IMF}_{\left(L+1\right)}\left(n\right)=\frac{1}{K}\sum _{k=1}^{K}{E}_{L}\left\{{C}_{L}\left(t\right)+{\sigma }_{L}{E}_{L}\left({\omega }_{k}\left(t\right)\right)\right\}$$

Finally, the $n$ steady-state IMFs are reconstructed to produce the original signal as

10 $$x\left(t\right)=C\left(t\right)+\sum _{m=1}^{M}\overline{{IMF}_{m}\left(t\right)}$$

The total number of IMFs of a time series signal including the residual is given as ${\text{log}}_{2}N$ where $N$ is the number of observations.

After decomposing the realised skewness and realised kurtosis values for all the cryptocurrencies, we then deploy the DY framework to examine the connectedness of the cryptocurrencies across time and different investing horizons (represented by the various IMFs). The DF framework utilises the generalised forecast error variance decomposition (GFEVD) in a VAR framework in place of utilising Cholesky factorisation. Using the DY (2012) framework, a covariance stationary $VAR\left(m\right)$ model is given by:

11 $${\text{Z}}_{t}=\sum\limits_{j-1}^{m}\mathfrak{I}{\text{Z}}_{t-j}+{\upsilon }_{t}$$

${\text{Z}}_{t}$ is the $n\times 1$ vector of observed variables at time $t$; $\mathfrak{I}$ is the $n\times n$ coefficient matrix and ${\upsilon }_{t}$ represent a matrix of serially uncorrelated error terms. Using this approach and assuming a covariance stationary VAR system, a representation of the moving average (MA) is then given as follows:

12 $${\text{Z}}_{t}=\sum\limits_{j-1}^{m}\mathfrak{I}{\text{Z}}_{t-j}+{\upsilon }_{t}$$

Where $n\times n$ coefficient matrix ${{\Lambda }}_{j}$ follow a recursive process ${{\Lambda }}_{j}={\psi }_{1}{{\Lambda }}_{j-1}+{\psi }_{2}{{\Lambda }}_{j-2}+\dots +{\psi }_{m}{{\Lambda }}_{j-m}$ with ${{\Lambda }}_{0}$ being the $n\times n$ identity matrix and ${{\Lambda }}_{j}=0$ for $j<0$.

The DY (2012) framework depends on the GFEVD framework which was formalised by Koop et al. (1996) and Pesaran and Shin (1998) to eradicate the effect of VAR ordering on the variance decomposition. Using this framework, H-ahead GFEVD is shown as:

13 $${\tilde{\pi }}_{ij,t}^{l}\left(H\right)=\frac{\sum _{t=1}^{H-1}{\mathfrak{I}}_{ij,t}^{2,l}}{\sum _{j=1}^{n}\sum _{t=1}^{H-1}{\mathfrak{I}}_{ij,t}^{2,l}}$$

With $\sum _{j=1}^{n}{\tilde{\pi }}_{ij,t}^{n}\left(H\right)=1$ and $\sum _{ij=1}^{n}{\tilde{\pi }}_{ij,t}^{n}\left(H\right)=n$. ${\mathfrak{I}}_{ij,t}^{2,l}$ represents the generalised impulse functions and ${\tilde{\pi }}_{ij,t}^{l}\left(H\right)$ shows the generalised forecast error variance decomposition. The total connectedness index is then extracted from the GFEVD to represent the interdependence among the variables as follows:

14 $${C}_{t}^{l}\left(H\right)= \frac{\sum _{ij=1,i\ne j}^{n}{\tilde{\pi }}_{ij,t}^{n}\left(H\right)}{\sum _{ij=1}^{n}{\tilde{\pi }}_{ij,t}^{n}\left(H\right)}\times 100$$

#### BK frequency connectedness framework

To measure the frequency dynamics of higher moment connectedness among sampled cryptocurrencies resulting from heterogeneous frequency responses to shocks in each of the cryptocurrencies, we also use the Baruník and Křehlík (2018)’s framework. This framework estimates the connectedness in short, medium and long-term cycles by utilising the spectral representation of variance decompositions. The frequency-domain connectedness measure of Baruník and Křehlík (2018) builds on the Diebold and Yilmaz (2012) connectedness measure which is based on the generalised forecast error variance decomposition (GFEVD). In the empirical analysis, we focus on the frequency bands up to 1 week (or 5 days), monthly bands (20 days) and yearly bands (250 days) together corresponding to the short-term, medium-term and long-term in line with other studies (e.g. Baruník and Křehlík 2018). Using this framework, when connectedness is created at high frequencies, this implies that in the underlying period, investors are processing information more quickly and that a higher moment shock to a specific cryptocurrency will only last in the short term. By the same virtue, the creation of higher moment connectedness at lower frequencies signifies that shocks are persistent and lasting for longer periods. We define the frequency connectedness on band $d$, where:

15 $${S}_{d}^{F}=\left\{\genfrac{}{}{0pt}{}{d=\left\{{a1}_{,}{a}_{2}\right\}}{{a}_{1},{a}_{2}\in \left[-\pi ,\pi \right],{a}_{1}<{a}_{2}}\right.$$

16 $${S}_{d}^{F}=100\left(\frac{\sum _{j\ne k}(\sim{{\Theta }}_{d}{)}_{j,k}}{\sum (\sim{{\Theta }}_{\infty }{)}_{j,k}}-\frac{Tr\{\sim{{\Theta }}_{d}\}}{\sum (\sim{{\Theta }}_{\infty }{)}_{j,k}}\right)$$

The band $d^{\prime }s$ within-frequency connectedness is as follows:

17 $${S}_{d}^{W}=100 \frac{Tr\{\sim{{\Theta }}_{d}\}}{\sum (\sim{{\Theta }}_{d}{)}_{j,k}}$$

Where generalised forecast error variance decomposition (FEVD) on different frequency bands $d$ is specified as:

18 $$\sum (\sim{{\Theta }}_{d}{)}_{j,k}=\frac{1}{2\pi }{\int }_{d}{\varvec{\Gamma }}_{\varvec{j}}\left(\omega \right)(f\left(\omega \right){)}_{j,k}d\omega$$

and

19 $${\varvec{\Gamma }}_{\varvec{j}}\left(\omega \right)=\frac{\left(\psi \right({e}^{-iw})\sum {\psi}^{{\prime }({e}^{+iw)}}{)}_{j,j}}{\frac{1}{2\pi }{\int }_{-\pi }^{\pi }(\psi \left({e}^{-i \lambda}\right)\sum {\psi }^{{\prime }\left({e}^{+i \lambda}\right)}{)}_{j,j}d \lambda}$$

is the weighting function, while the frequency response function is defined as:

20 $$\psi \left({e}^{-iw}\right)=\sum _{h}{e}^{-iwh}{\psi }_{h}$$

## Empirical results

**Fig. 1:**
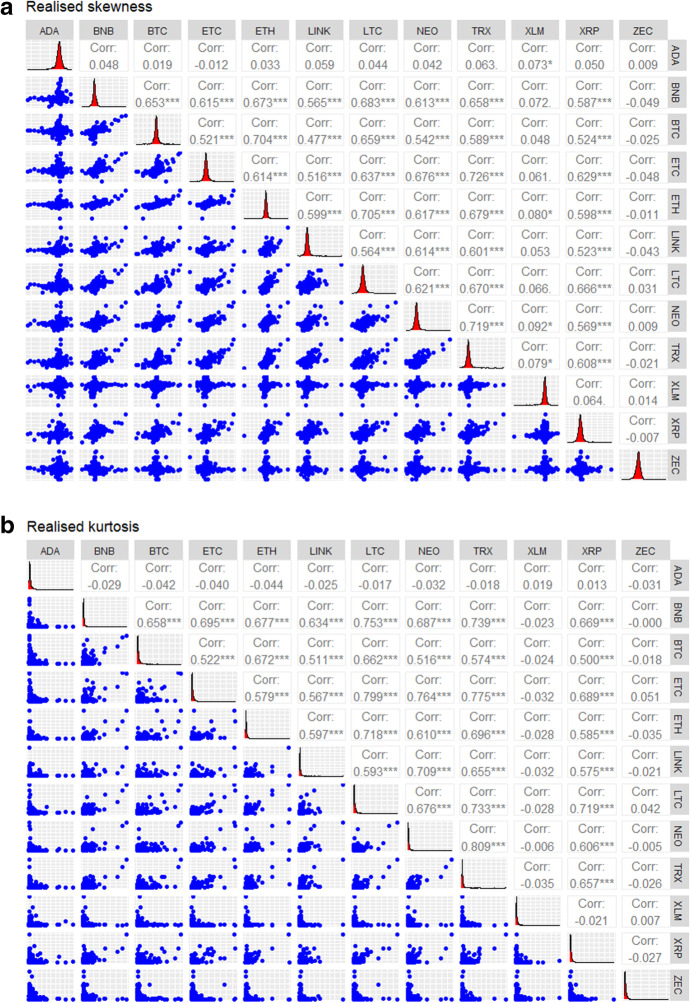
**A **Pairwise Pearson correlation matrix for realised skewness. **B** Pairwise correlation matrix for realised kurtosis. Notes: *,**,*** shows that the Pearson correlations are statistically different from zero at the 10%, 5% and 1% levels of significance

In this section, we report the findings of the connectedness among cryptocurrencies using the different econometric models adopted. We first report the descriptive statistics displayed in Table 1. From Table 1, it can be observed that the realised skewness and realised kurtosis for all cryptocurrencies are not normally distributed as the Jarque Bera test statistics are all statistically significant at the 1% level leading to the rejection of the null hypothesis of normal distributions. It can also be observed that the Augmented Dickey-Fuller test statistics are statistically significant at the 1% level for the realised skewness and realised kurtosis for all the sampled cryptocurrencies, again leading us to the rejection of the null hypothesis of the presence of a unit root in the series, signifying that the time variables are stationary. The deviation of the variables from a Gaussian distribution as seen from the Jarque Bera test signifies that the utilisation of parametric models to examine the relationships among the variables is likely to lead to biased results and model misspecification. This further justifies the use of the models used in the present study which are non-parametric and robust in the presence of non-linearities and stylised facts of financial variables. Figure 1A and B visualise the Pairwise Pearson Correlations for realised skewness and realised kurtosis respectively. Correlations are shown in the upper triangles.

**Table 1:** Preliminary statistics

Realised skewness						Realised kurtosis				
	Mean	Min	Max	JB	ADF	Mean	Min	Max	JB	ADF
ADA	-0.0717	-8.3267	3.4388	7613***	-9.2089***	8.0894	2.8343	121.8967	109,686***	-8.508***
BNB	0.2356	-4.9497	12.8258	7613***	-7.5816***	8.6895	2.8897	198.5068	282,972***	-7.7397***
BTC	-0.0046	-11.2596	13.0564	7699***	-9.2474***	12.4824	2.9483	203.6198	53,989***	-6.9447***
ETC	0.1233	-6.9801	12.1296	14,943***	-8.2761***	10.2493	1.8517	186.5635	114,255***	-8.3972***
ETH	0.0400	-13.8669	13.7445	48,831***	-9.8929***	9.32148	2.6786	221.8918	424,490***	-6.9211***
LINK	0.0761	-3.6537	13.6083	54,303***	-7.7983***	7.6079	2.5511	214.5452	807,916***	-7.7647***
LTC	0.0748	-4.2267	12.5986	16,288***	-8.7172***	9.6318	2.7860	193.8749	216,581***	-7.5429***
NEO	0.1122	-3.9385	12.5815	27,125***	-7.8429***	8.6943	2.8973	193.2646	401,121***	-8.6079***
TRX	0.3252	-3.1613	14.0071	21,150***	-8.8579***	9.4250	2.8717	222.8421	236,429***	-8.1674***
XLM	-0.1607	-13.7872	5.6555	27,423***	-8.1382***	8.9335	2.8061	218.9901	399,347***	-8.446***
XRP	0.1780	-5.5353	13.9832	9324***	-8.3683***	10.8589	3.0722	222.9855	138,188***	-8.1509***
ZEC	-0.0179	-5.3381	8.7450	7084***	-8.9829***	8.5173	2.5697	129.8931	106,340***	-8.4981***

Notes: Table 1 shows the preliminary statistical properties of the realised skewness and the realised kurtosis for each cryptocurrency. All the statistics are presented at daily granularity. Mean, Min and Max represent the average, minimum value and maximum value respectively, while JB and ADF represent the Jarque Bera test statistics and the Augmented Dickey-Fuller test statistics. *** shows statistical significance at the 1% level.

**Fig. 2:**
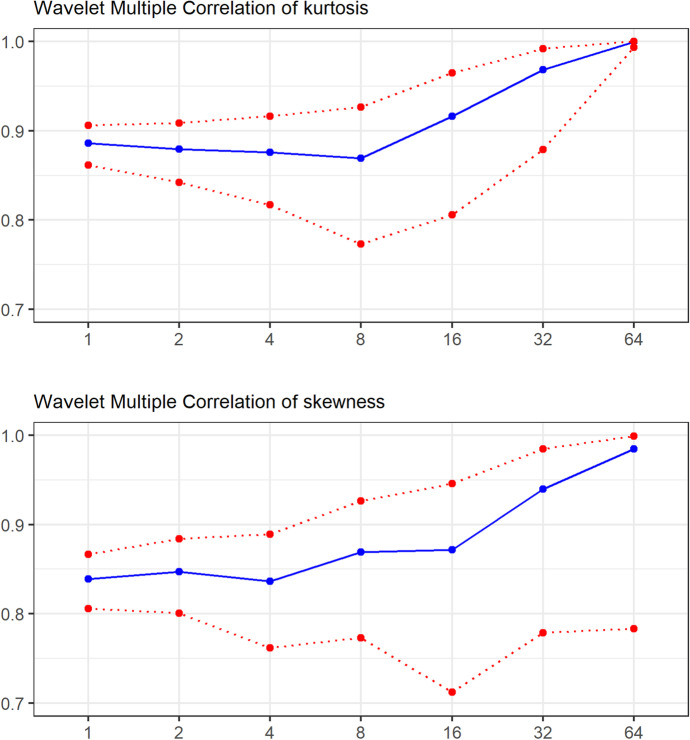
Connectedness from multiple wavelet correlations. Notes: The blue continuous lines show the multiple correlation coefficient (represented by the vertical axes) at different scales (represented by the horizontal axes) while the red dotted lines show the upper and lower 95% confidence bands

As shown in Fig. 1A and B, the higher moment correlation coefficients among the cryptocurrencies are mostly positive and more than 0.5. ETC and LTC (0.799) are the most correlated in terms of realised kurtosis while ETC and TRX (0.726) are the most correlated in terms of realised skewness. Figure 1A and B, therefore, provides us with preliminary evidence of statistically significant higher moment connectedness among the cryptocurrencies which warrants a formal investigation using robust econometric models. The scatter plots also show the presence of significant outliers which calls for the utilisation of methods that are robust in the presence of outliers and non-stationary data. In Fig. 2, we present the findings on higher moment connectedness using Wavelet Multiple Correlations.

The results in Fig. 2 shows that the Wavelet Multiple Correlation for both the realised kurtosis and the realised skewness is high across all the time scales, with the minimum values for both moments above 0.8. Also, it can be noted that 95% confidence bands for kurtosis are narrow at the lowest and highest time scales while wider at the middle of the scale distribution. For kurtosis, the 95% confidence bands are narrower at lower time scales and increase with the increases in the time scales. Lower frequency components of the wavelet analysis represent longer-term trends in the data. It is possible that the skewness of longer-term trends is more difficult to estimate accurately due to changes in market conditions, investor sentiment, and other external factors that affect cryptocurrency prices. This could result in higher variability and wider confidence bands for skewness estimates at lower frequencies. Some studies (e.g. Jiang et al., 2018) have provided empirical evidence that shows that the cryptocurrency market exhibits persistence in its returns, such as long periods of bullish or bearish sentiment, and this can lead to a higher degree of kurtosis and narrower confidence bands for realised kurtosis estimates at lower frequencies In terms of the evolution of the moments across different time scales, the WMC coefficients are almost constant for the first four time scales for kurtosis while for skewness the WMC coefficients are almost constant for the first five time scales. This shows that at most of the higher frequencies, investors are affected homogenously by higher moments and heterogeneity is only seen at the highest time scales. Figure 2 shows a clear tendency for the contemporaneous multiple correlations to increase as the time horizon increases. This is in line with studies done in the first two moments that show that diversification benefits are more prevalent in the short and medium runs (e.g. Omane-Adjepong and Alagidede 2019).

**Fig. 3:**
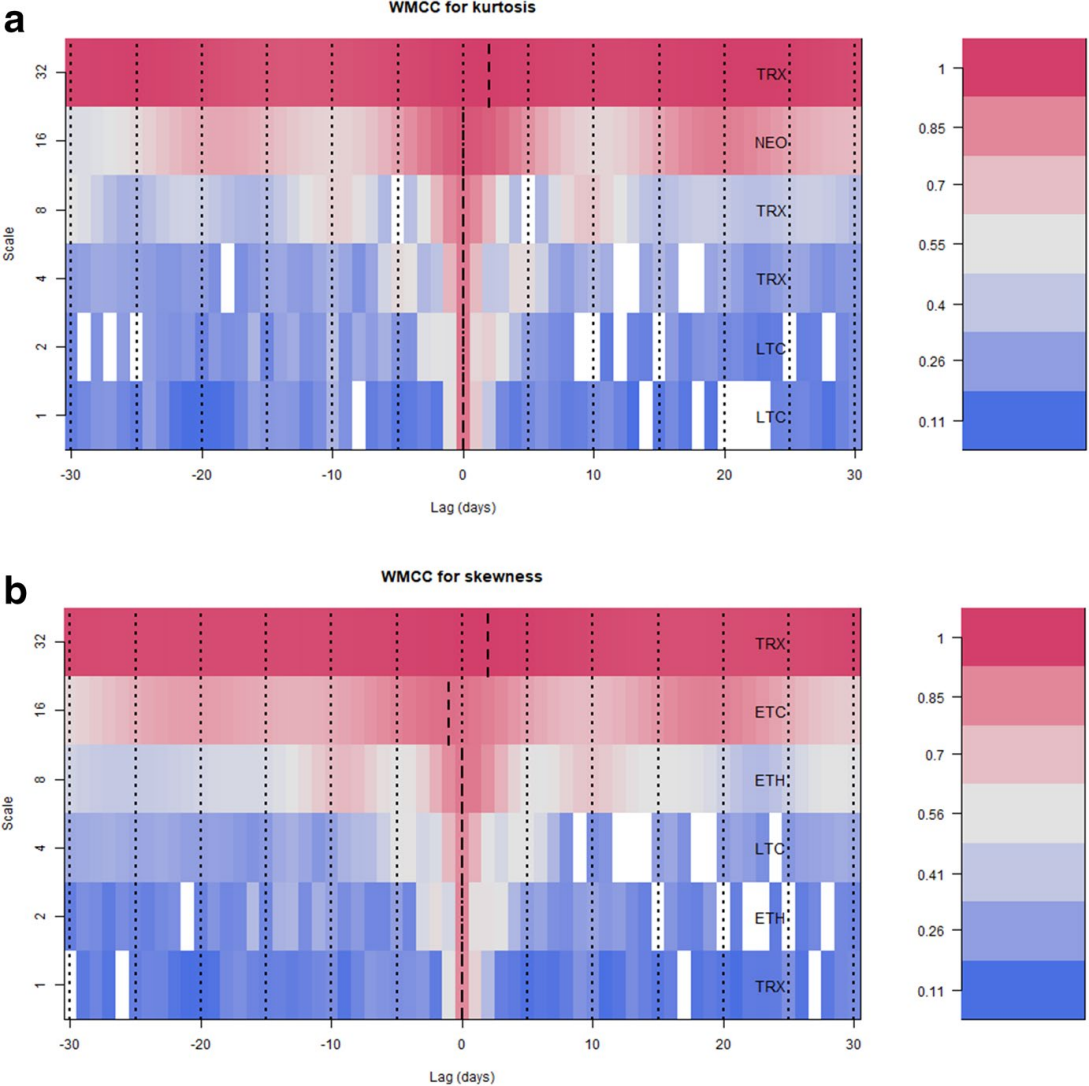
**A** Findings from wavelet multiple cross-correlations-realised kurtosis. **B** Findings from wavelet multiple cross-correlations-realised skewness. Notes: Figure **A** and **B** show the wavelet multiple cross-correlations for the different wavelet scales using leads and lags of 30 trading days. To the right of the plot is a list of cryptocurrencies that maximise the multiple correlations against a linear combination of the rest of the cryptocurrencies and shows a systemwide potential leader or follower

The results for the two moments visualised in Fig. 2 show obvious discrepancies at lower frequencies showing that the differences among these cryptocurrencies in terms of the respective higher moments may portray the actuation of distinct agents with different investing horizons. Previous studies (Hasan et al. 2021; Bouri et al. 2022) have reported a near-perfect integration among cryptocurrencies at longer investing horizons at the return and volatility levels. This study shows further evidence of this near-perfect integration of cryptocurrencies at the level of higher moments of cryptocurrency return distributions. Figure 3A and B wavelet multiple cross-correlation results to identify the leaders and followers in the transmission and reception of higher-order shocks within the cryptocurrency market.

Figure 3A and B demonstrate that both skewness and kurtosis show similar patterns in their wavelet multiple cross-correlations for various cryptocurrencies. The study shows that as the investment horizon increases, the cross-correlations among cryptocurrencies become stronger, as depicted by the shift from blue to dark red colours from the lowest scales to the highest scales. In particular, for kurtosis, LTC has the potential to lead or lag at the highest frequency scales of 1 and 2, whereas TRX and NEO have the potential to lead and lag at medium-term frequency scales of 3 and 4. At the lowest frequency, the study shows that there is significant evidence that TRX lags in the cross-correlation of kurtosis among the selected cryptocurrencies. As for skewness, TRX, ETH, and LTC, all have the potential to lead and lag for scales up to 8, while ETH is a clear leader at scale 16, and TRX lags at the highest scale (lowest frequency). This information can be useful for investors to identify potential leaders and followers among cryptocurrencies at different scales, allowing them to make informed investment decisions. Notably, our results differ from existing literature that uses the first two moments that has reported the most influential cryptocurrencies being Bitcoin (Kumar and Ajaz 2019; Qiao et al. 2020; Kyriazis 2019), Ripple and Ethereum (Qureshi et al. 2020). However, these results seem to be in line with Li et al. (2020) who reported that small-cap cryptocurrencies tend to transmit risks to large-cap cryptocurrencies.

In Table 2, we present the CEEDMAN-based DY (2012) connectedness using 5 IMFs. As shown in Table 2, the mean, minimum and maximum values all show an increasing pattern with the IMFs. There is evidence that higher-moment connectedness increases with the increases in the IMFs.

**Table 2:** CEEMDAN-based DY connectedness

Realised skewness				Realised kurtosis		
IMF	Mean	Min	Max	Mean	Min	Max
1	48.68	31.73	68.47	50.97	29.89	91.66
2	56.22	45.05	91.57	56.64	33.39	92.96
3	67.32	53.89	88.17	64.64	49.65	91.66
4	82.73	73.72	92.04	86.77	80.46	91.47
5	90.72	83.16	94.35	90.72	83.15	94.35

**Fig. 4:**
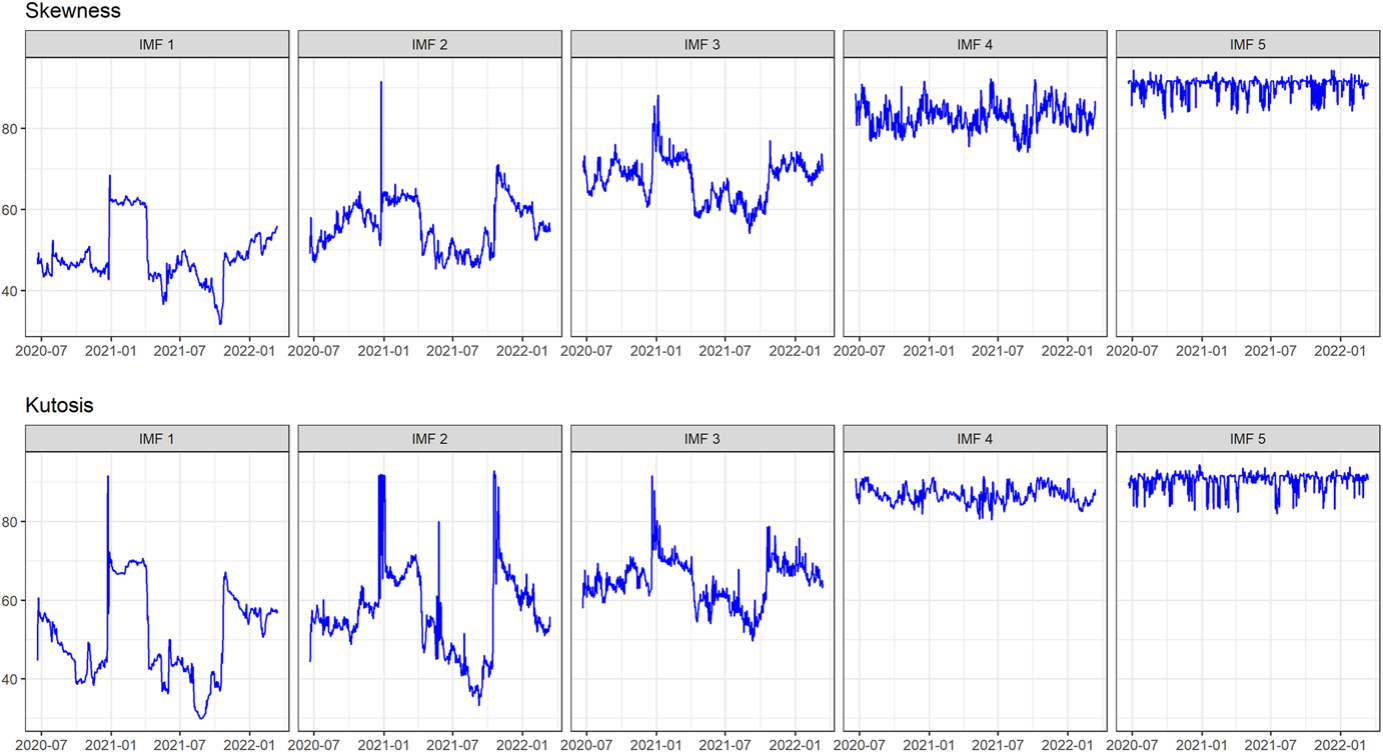
CEEMDAN-based DY framework

The same pattern that is depicted in the descriptive statistics in Table 2 can be observed in Fig. 4. The higher moment time-varying connectedness of both skewness and kurtosis seems to exhibit an upward trend and is also cyclical. This shows that the higher moment connectedness among the 12 sampled cryptocurrencies is higher at lower frequencies (higher IMFs). Another notable trend that can be observed in the dynamics of higher moment connectedness is the unambiguous spike in connectedness around the beginning of 2021. In 2021, several countries, including China and India, announced plans to regulate cryptocurrencies more closely. This created uncertainty and volatility in the cryptocurrency markets, as investors were unsure how these regulatory changes would affect the market. Also, cryptocurrencies gained more mainstream acceptance in 2021, with companies like Tesla and PayPal^1^ announcing plans to accept cryptocurrencies as payment. This increased acceptance led to greater demand for cryptocurrencies, driving up prices and volatility. The unique characteristics of cryptocurrencies, such as their decentralised nature and lack of regulation, make them particularly prone to spikes in skewness and kurtosis during uncertain periods.

**Fig. 5:**
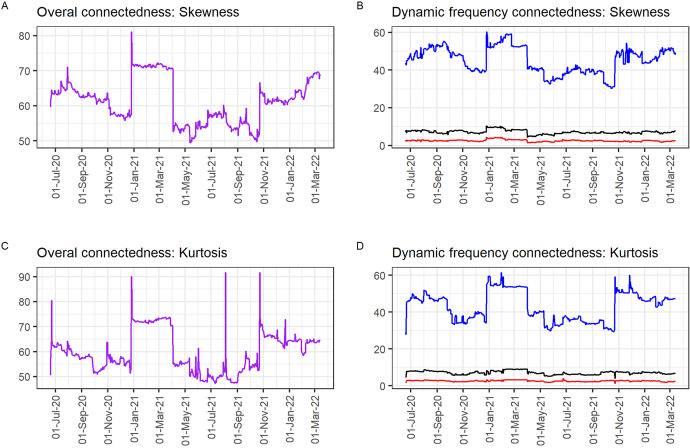
Results from the BK frequency connectedness framework

In further robustness checks, we report the time-frequency higher-order connectedness of the cryptocurrencies using the BK frequency connectedness framework, results which are visualised in Fig. 5. In Fig. 5, Panels A and C show the overall connectedness of the realised skewness and realised kurtosis respectively. Panels B and D show the dynamic frequency connectedness of skewness and kurtosis with the different colours representing the following frequency bands respectively: blue > long-term, black > medium-term, and red > short-term.

The results in Fig. 5 show that for both realised skewness and realised kurtosis, long-term dynamics are contributing more to the overall connectedness among the cryptocurrencies followed by the short-term and medium-term dynamics respectively. The results corroborate the results from the Wavelet Multiple Correlation as well as the CEEMDAN-based DY framework which reveal higher connectedness at lower frequencies. This shows that higher moment spillovers among cryptocurrencies is persistent and long-lasting. The results also echo the suggestion that connectedness increases in turbulent times (e.g. Bouri et al. 2021) as seen in the spikes in higher-moment connectedness amid the uncertainty mostly from the promises to regulate the market.

The results from wavelet multiple correlations, the CEEMDAN-based DY framework and the BK framework unanimously point to higher moment connectedness among the sampled cryptocurrencies, with the connectedness amplified at lower frequencies. This means that there are higher chances of cryptocurrencies crashing together and also experiencing extreme returns (both positive and negative returns) together. This provides evidence of the existence of herding behaviour in the crypto markets as documented by Vidal-Tomás et al., (2019). The findings pointing to the cryptocurrencies experiencing extreme returns at the same time are in line with the co-explosivity phenomena (Bouri et al. 2019) and tail risk dependence phenomena (Nguyen et al. 2020).

## Conclusion

Cryptocurrency has become an important asset class in the past few years. A lot of research has been done to untangle different facets of this growing asset class. The majority of the research has however mostly concentrated on the first two moments (return and volatility) of cryptocurrencies and has left the higher moment properties of the cryptocurrencies at the peripherals. We particularly investigate the higher moment (realised skewness and realised kurtosis) connectedness of 12 cryptocurrencies sampled at high frequency (1-minute intervals) in a time-frequency domain. We apply methods robust in the presence of nonlinearities, deviation from Gaussian distributions and several stylised facts of financial variables. Our results show high moment connectedness of cryptocurrencies which is mostly amplified at lower frequencies. These results have important implications for policymakers and investors. For policymakers, the findings suggest that they need to consider the higher moment connectedness when developing regulatory frameworks for the cryptocurrency market. This may involve developing new risk management strategies that specifically target higher-moment shocks to the system, as well as improving the monitoring and surveillance of the market to better detect and respond to such shocks. For investors, the findings suggest that they need to be aware of the potential for higher moment shocks in the cryptocurrency market and should consider incorporating this information into their investment strategies. This may involve using more sophisticated risk management techniques that take into account the higher moment correlations between different cryptocurrencies, as well as carefully monitoring the leading and lagging indicators identified in the study.

## Data Availability

The datasets generated during and/or analysed during the current study are available from the corresponding author on reasonable request.

## Footnotes

1
https://www.cnbc.com/2021/02/08/tesla-buys-1point5-billion-in-bitcoin.html.
